# Dose patterns in commercially insured subjects chronically exposed to opioids: a large cohort study in the United States

**DOI:** 10.1186/1472-684X-9-14

**Published:** 2010-06-18

**Authors:** Maria Soledad Cepeda, Mila Etropolski, Rachel Weinstein, Daniel Fife, Raymond Boston, Amy Matcho

**Affiliations:** 1 Johnson & Johnson Pharmaceutical Research and Development, Titusville, NJ, USA; 2 University of Pennsylvania. School of Veterinary Medicine, Department of Clinical Studies, NBC, Philadelphia, PA, USA

## Abstract

**Background:**

Little data exist on how opioid doses vary with the length of exposure among chronic opioid users.

**Methods:**

To characterize the change in the dosage of opioids over time, a retrospective cohort study using the PharMetrics database for the years 1999 through 2008 was conducted. Individuals exposed to opioids in 2000 who had 2 opioid dispensings at least 6 months apart and were opioid naive (did not receive any opioid 6 month before their exposure in 2000) were included. The date of the first dispensing in 2000 was defined as the index date and the dispensing had to be for a strong and full agonist opioid. All opioid doses were converted to oral morphine equivalent doses. Exposure was classified as continuous or intermittent. Mean, median, interquartile range, and 95^th ^percentile of opioid dose over 6-month periods, as well as the percentage of subjects who ever received a high or very high opioid dose, were calculated.

**Results:**

Among the 48,986 subjects, the mean age was 44.5 years and 54.5% were women. Intermittent exposure was observed in 99% of subjects; continuous exposure was observed in 1% of subjects. The mean duration of exposure for the subjects who were continuously exposed to opioids was 477 days. In subjects with no cancer diagnosis who were continuously exposed to opioids, the mean, 25^th^, 50^th^, and 75^th ^percentile of dose was stable during the first 2 years of use, but the 95^th ^percentile increased. Seven percent of them were exposed to doses of 180 mg or more of morphine at some point.

**Conclusions:**

Dose escalation is uncommon in subjects with intermittent exposure to opioids. For subjects with continuous exposure to opioids who have cancer, doses rise substantially with time. For those without cancer, doses remain relatively stable for the first 2 years of use, but subsequently increase. Seven percent of subjects with no cancer diagnosis will be exposed to daily doses of 180 mg or more of morphine equivalent at some point.

## Background

Opioids are increasingly used for the treatment of chronic malignant and nonmalignant pain [1,2] and systematic reviews of randomized controlled trials have confirmed their short-term efficacy for the treatment of neuropathic pain, back pain, ostearthritis, cancer pain, and fibromyalgia [3-8].

However, in some cases with chronic use, the dose of opioids may increase because of disease progression, the development of tolerance and/or the development of a state of abnormal high pain sensitivity (hypersensitivity) that opioids themselves may induce. Hypersensitivity is a poorly understood phenomenon thought to result from opioid-induced neuroplastic changes in the peripheral and central nervous systems that lead to sensitization of pronociceptive pathways [1,9].

Little data exist to support the long-term efficacy of opioids or to describe the relation between opioid dose and the length of exposure among chronic opioid users [10]. Although randomized controlled trials have evaluated opioids for the treatment of chronic pain, most of these trials have limited follow-up periods (around 16 weeks) [3-5] and in the trials with longer follow-up periods, the lack of generalizability of the findings has been identified as a serious shortcoming [4].

Claims databases provide an opportunity to close this gap in knowledge. These databases are a collection of health insurance claims that are maintained largely for billing and administrative purposes. Nevertheless, they permit the evaluation of not only a diverse population, but also a large number of subjects followed over a relatively long period of time in a real-world setting [11].

Health care databases have been used extensively for pharmacoepidemiologic research in many therapeutic areas including pain [12-14] to describe health care utilization, patterns of care, disease prevalence, drug and disease outcomes, and cost of care. There are, however, limitations to the use of health care databases for pharmacoepidemiologic research: they are observational, which limits inferences about treatment efficacy relative to studies that include random allocation to treatment [11]; they may not include information on important confounding factors (eg, smoking), and they may include diagnoses that are provisional or whose selection may be affected by reimbursement policies. The advantages to the use of these databases are the availability of systematic and accurate information on prescribed medications [11], their ability to follow patients for many years, and the fact that they reflect clinical practice in a population that is not subject to the same selection biases as might apply to those who volunteered for inclusion in a study. In fact, health care databases often are used to explain differences in findings between trial data and clinical practice [15].

We sought to characterize the dose of opioids in both cancer and noncancer patients intermittently and chronically exposed to opioids using PharMetrics Patient-Centric database. PharMetrics is the largest health care claims database in the United States and is representative of the commercially insured population. Data for more than 61 million individuals from more than 98 health plans across the United States are available and include information on all hospitalizations, emergency care, office visits, and drug dispensing covered by insurance.

## Methods

To characterize the change in the dose of opioids over time, a retrospective cohort study using the PharMetrics Patient-Centric database for the years 1999 through 2008 was conducted. Access to the PharMetrics database requires a license agreement and the data are provided de-identified. Tabulations from these data do not require ethics approval.

### Inclusion and exclusion criteria

Opioid-naive individuals exposed to opioids in 2000 who had 2 strong opioid dispensings at least 6 months apart (to focus on subjects with long-term opioid exposure), regardless of the duration of the prescription, were included. Opioid-naive individuals were defined as subjects who did not receive any type of opioid for at least 6 months before their first opioid dispensing in 2000.

The date of the first dispensing of a strong opioid prescription in 2000 was defined as the index date. The dispensing at the index date had to be for a strong and full agonist opioid (e.g., morphine, hydromorphone), Table 1. Oral, rectal, transdermal, subcutaneous, intramuscular or intravenous routes of administration and all forms of presentation (immediate release or controlled release) were included. A subject remained in the cohort even if after receiving a strong opioid at the index date, he or she subsequently received a weak, agonist antagonist, or partial agonist opioid. All opioid doses were converted into oral morphine equivalent doses.

**Table 1:** Strong opioids dispensed at index date

Opioid medication	Percentage
Fentanyl	0.2

Hydrocodone	78.8

Hydromorphone	0.2

Meperidine	1.7

Methadone	0.004

Morphine	0.2

Oxycodone	18.7

Oxymorphone	0.002

*Percentages may not add up to 100% because of rounding.

Patients who were receiving opioids for the treatment of opioid addiction were excluded. To determine presence of addiction before the index date, the International Statistical Classification of Diseases and Related Health Problems, ninth edition (ICD-9) diagnostic codes for drug dependence or drug abuse were used.

### Pattern of exposure

Exposure was classified as either continuous or intermittent. A subject was defined as "continuously exposed" if there were no time gaps between the dispensing of opioids, and "intermittently exposed" if there was a time gap between the dispensing of opioids. A gap was considered to occur when the number of days between 2 dispensings was more than 4 times the number of days supplied by the previous dispensing. Four times the days supplied was used to take into account that some patients may have taken the medication less often than prescribed.

### Daily dose and dose over time

Daily dose was calculated from the quantity dispensed and the days supplied. Daily doses were re-expressed as oral morphine equivalents using the conversion factors shown in Table 2.

**Table 2:** Morphine equivalent conversion factors

Opioid drug	Oral morphine equivalent dose
Buprenorphine	Multiplied by 60

Butorphanol	Multiplied by 7

Codeine	Divided by 12

Dihydrocodeine	Divided by 10

Fentanyl (patch)	Multiplied by 150

Hydrocodone	Multiplied by 1.5

Hydromorphone (oral)	Multiplied by 5

Hydromorphone (parenteral)	Multiplied by 10

Levorphanol	Multiplied by 6

Meperidine	Divided by 12

Methadone	Multiplied by 10

Morphine (parenteral)	Multiplied by 2

Oxycodone	Multiplied by 1.5

Oxymorphone	Multiplied by 10

Pentazocine	Divided by 4

Propoxyphene	Divided by 24

Tramadol	Divided by 5

To determine the behavior of opioid dose over time, mean, median dosage, interquartile range and 95^th ^percentile of opioid dose over 6-month periods from the index date were calculated. Opioid doses are reported separately for subjects with intermittent or continuous exposures and, for subjects with continuous exposure, by the presence or absence of a cancer diagnosis.

To address the possibility that aggregate dose changes at the cohort level might be affected by selective loss of the subjects with the highest or lowest doses, in each 6-month period, the dose of opioids for subjects who remained in the cohort during all or part of the next 6-month period were examined separately from subjects who, for any reason, did not continue to the next 6-month period.

The database does not explicitly link medication dispensings to their indications. To assess the likely indications for the opioids that were dispensed, ICD-9 pain-related diagnostic codes were grouped into cancer, musculoskeletal, migraine, neuropathic, and other. Because many opioid dispensings did not have an ICD-9 pain-related diagnosis that was close in time, no time limitation was placed on these pain diagnoses.

The percentage of subjects who ever received a high or very high opioid dose was also calculated. Morphine equivalents of 180 mg/day or more were considered as the high dose category, and morphine equivalents of 300 mg/day or more were considered as the very high dose category [1,16]. In addition, the percentage of subjects who ever received morphine equivalents of 100 mg/day or more was also calculated as such doses have been associated with an increase risk of overdose [17].

The analyses were performed in STATA IC version 10.1.

## Results

A total of 57,345 subjects were exposed to opioids starting in 2000 for at least 6 months and met the inclusion criteria. Of these, 8,362 (14%) subjects were excluded because of missing data on the quantity dispensed or the days supplied, leaving 48,986 subjects whose dosage patterns were examined in the present study. Among these subjects, the mean age was 44.5 years and 54.5% were women. Subjects were diagnosed with various possible types of pain (some subjects were diagnosed with more than one type), including musculoskeletal, 77.6%; neuropathic, 35.3%; migraine, 27.8%; and cancer, 24.2%.

At the index date, the most frequently dispensed opioid was hydrocodone (78.8% of subjects), followed by oxycodone (18.7% of subjects) and meperidine (1.7% of subjects). Fentanyl, hydromorphone, methadone, morphine, and oxymorphone together accounted for the remaining 0.8% of dispensing at the index date (Table 1).

### Pattern of exposure: Intermittent and continuous

Intermittent exposure was observed among 48,367 (99%) of subjects; continuous exposure was observed among 619 (1%) of subjects. The median number of opioid dispensings was 5 for subjects with intermittent exposure (range, 2-319 dispensings) and 13 for subjects with continuous exposure (range, 2-275 dispensings). The mean duration of exposure in the subjects continuously exposed to opioids was 477 days (range, 6 months to 8 years).

The median daily dose of morphine equivalent in the subjects with intermittent exposure remained stable over time, at approximately the equivalent of 50 mg oral morphine per day. The mean (approximately 60 mg), twenty-fifth percentile (approximately 30 mg), seventy-fifth percentile (approximately 60 mg) and ninety-fifth percentile (approximately 120 mg) doses were also stable over time (Table 3).

**Table 3:** Morphine equivalent daily dose by time in subjects with intermittent exposure

Years	Number of subjects	Mean ± SD	Median	Min	Max	P25	P75	P95
Half year	48367	62.48 ± 76.43	50.00	0.25	2700.00	37.50	69.17	112.50

> 6 months to 1 year	22494	57.61 ± 85.68	45.00	0.33	6666.67	31.50	65.16	112.50

> 1 to 1.5 years	19130	56.07 ± 69.1	45.00	0.50	2250.00	30.00	64.11	112.50

> 1.5 to 2 years	15553	56.97 ± 73.8	45.00	0.50	2025.00	30.00	65.00	112.50

> 2 to 2.5 years	11588	58.45 ± 69.05	46.15	0.67	1920.00	30.00	67.50	113.44

> 2.5 to 3 years	8119	60.73 ± 82.99	46.88	0.67	1920.00	30.94	67.50	120.00

> 3 to 3. 5 years	5824	58.23 ± 81.27	45.00	1.56	2660.63	30.00	64.29	112.50

> 3. 5 to 4 years	4677	55.71 ± 75.7	45.00	2.50	1808.04	30.00	60.00	112.50

> 4 to 4.5 years	4619	58.57 ± 87.44	45.00	2.50	2025.00	30.00	60.00	120.00

> 4.5 to 5 years	4111	59.42 ± 100.09	45.00	2.50	2700.00	30.00	60.00	112.50

> 5 to 5.5 years	3418	58.56 ± 96.34	45.00	2.50	3535.12	30.00	60.00	120.00

> 5.5 to 6 years	3301	62.96 ± 113.2	45.00	2.50	2890.19	30.00	61.25	120.00

> 6 to 6.5 years	3225	61.66 ± 109.87	45.00	2.45	3368.28	30.00	62.00	120.00

> 6.5 to 7 years	3031	61.81 ± 101.48	45.00	2.50	2239.86	30.00	60.99	114.28

> 7 to 7.5 years	1837	64.44 ± 116.78	45.00	3.75	2695.92	30.00	60.00	128.57

> 7.5 to 8 years	714	62.71 ± 103.76	45.00	4.17	1350.00	30.00	56.25	146.93

> 8 years	179	72.52 ± 124.42	46.32	6.94	937.50	28.75	60.00	300.00

SD, standard deviation; P25, 25^th ^percentile; P75; 75^th ^percentile; P95; 95^th ^percentile

Six hundred and nineteen subjects were continuously exposed to opioids for at least 6 months, and 6 years after the index day, only 9 subjects were continuously exposed to opioids. The daily morphine equivalent dose in subjects with continuous exposure and no cancer diagnosis remained stable for the first two years, as measured by mean (approximately 70 mg), median (approximately 50 mg), twenty-fifth (approximately 30 mg) or seventy-fifth percentiles (approximately 75 mg), but the 95^th ^percentile dose rose from 143 mg to 185 mg. After the second year of continuous exposure, although the median dose remained stable, the mean, seventy-fifth percentile and ninety-fifth percentile doses rose gradually. After the fourth year of continuous exposure the median opioid dose increased as well, though it should be noted that the number of subjects was small. The daily morphine equivalent dose in subjects with continuous exposure and a cancer diagnosis increased earlier than in subjects without cancer diagnosis (Tables 4 and 5).

**Table 4:** Morphine equivalent daily dose by time in subjects with continuous exposure without cancer diagnosis

Years	Number of subjects	Mean ± SD	Median	Min	Max	P25	P75	P95
Half year	442	62.53 ± 69.88	45.04	7.5	802.13	30.54	67.5	142.5

> 6 months to 1 year	305	62.11 ± 65.73	45	7.5	486.86	30	64.29	170

> 1 to 1.5 years	173	66.37 ± 64.39	46.61	7.5	480	30	73.5	197.14

> 1.5 to 2 years	107	64.6 ± 62.1	45.8	7.5	342.86	30	64.29	184.85

> 2 to 2.5 years	67	71.96 ± 70.86	50.63	8.33	360	30	82.12	183.33

> 2.5 to 3 years	29	88.2 ± 91.12	60	8.33	420	39.99	95.45	330

> 3 to 3. 5 years	19	91.05 ± 115.16	53.09	8.33	480	30	90.91	480

> 3. 5 to 4 years	14	68.13 ± 53.39	52.5	15	187.5	24.64	107.76	187.5

> 4 to 4.5 years	12	90.38 ± 79.83	52.5	15	244.55	30	169	244.55

> 4.5 to 5 years	9	80.99 ± 61.23	60	22.5	183	45	95.98	183

> 5 to 5.5 years	6	111.56 ± 72.04	117.18	22.5	187.5	45	180	187.5

> 5.5 to 6 years	6	122.59 ± 89.37	120.27	22.5	240	45	187.5	240

> 6 to 6.5 years	5	122.88 ± 105.75	75	22.5	255	45	216.92	255

> 6.5 to 7 years	5	133.5 ± 132.31	60	22.5	330	45	210	330

> 7 to 7.5 years	2	123.41 ± 110.89	123.41	45	201.82	45	201.82	201.82

> 7.5 to 8 years	2	127.5 ± 116.67	127.5	45	210	45	210	210

> 8 years	1	45 ± .	45	45	45	45	45	45

SD, standard deviation; P25, 25^th ^percentile; P75; 75^th ^percentile; P95; 95^th ^percentile

**Table 5:** Morphine equivalent daily dose by time in subjects with continuous exposure with cancer diagnosis

Years	Number of subjects	Mean ± SD	Median	Min	Max	P25	P75	P95
Half year	177	65.29 ± 67.61	50.00	7.50	699.57	33.88	77.73	140.63

> 6 months to 1 year	148	86.63 ± 156.56	55.44	7.50	1702.50	30.00	89.10	240.00

> 1 to 1.5 years	79	71.96 ± 86.73	51.46	10.56	560.00	26.25	79.29	294.49

> 1.5 to 2 years	39	90.96 ± 92.61	60.00	15.00	431.88	30.00	98.12	347.94

> 2 to 2.5 years	19	99.55 ± 87.31	75.00	15.00	300.00	37.50	120.00	300.00

> 2.5 to 3 years	9	116.79 ± 95.72	105.00	15.00	318.12	45.00	139.74	318.12

> 3 to 3. 5 years	5	121.9 ± 130.19	45.00	15.00	298.74	27.50	223.27	298.74

> 3. 5 to 4 years	5	115.78 ± 125.49	45.00	15.00	295.71	23.82	199.38	295.71

> 4 to 4.5 years	5	151.37 ± 182.88	45.00	15.00	436.67	26.25	233.96	436.67

> 4.5 to 5 years	4	304.47 ± 422.07	141.43	15.00	920.00	30.00	578.93	920.00

> 5 to 5.5 years	3	394.28 ± 457.61	240.00	33.75	909.09	33.75	909.09	909.09

> 5.5 to 6 years	3	400.01 ± 471.87	233.78	33.75	932.50	33.75	932.50	932.50

> 6 to 6.5 years	3	315.39 ± 321.23	240.00	38.57	667.61	38.57	667.61	667.61

> 6.5 to 7 years	2	187.59 ± 209.35	187.59	39.55	335.62	39.55	335.62	335.62

> 7 to 7.5 years	1	381.73 ± .	381.73	381.73	381.73	381.73	381.73	381.73

> 7.5 to 8 years	0	-	-	-	-	-	-	-

> 8 years	0	-	-	-	-	-	-	-

SD, standard deviation; P25, 25^th ^percentile; P75; 75^th ^percentile; P95; 95^th ^percentile

The opioid dose among subjects whose exposure ended in a given 6-month time period was similar to the opioid dose among subjects who remained exposed in the next 6-month time period (Table 6).

**Table 6:** Morphine equivalent median daily doses in subjects continuously exposed to opioids by time and by permanence in the cohort

Time period	Number of subjects who did not continue	Median dose in subjects who did not continue	Number of subjects who continued exposure	Median dose in subjects who continued exposure
Half year	166	56.25	453	45.0

> 6 months to 1 year	200	48.21	253	44.4

> 1 to 1.5 years	107	51.45	145	45.0

> 1.5 to 2 years	60	46.07	86	49.75

> 2 to 2.5 years	48	45.67	38	60.0

> 2.5 to 3 years	14	75.5	24	54.06

> 3 to 3. 5 years	5	60	19	52.2

> 3. 5 to 4 years	2	47.14	17	45.0

> 4 to 4.5 years	4	30.52	13	60.0

> 4.5 to 5 years	4	37.5	9	96.0

> 5 to 5.5 years	0	--	9	156.4

> 5.5 to 6 years	1	240.0	8	120.0

> 6 to 6.5 years	1	240.0	7	75.0

> 6.5 to 7 years	4	49.8	3	210.0

> 7 to 7.5 years	1	381.7	2	123.4

> 7.5 to 8 years	1	210.0	1	45.0

Median dose		56.25		45.0

### Exposure to high doses of opioids

In subjects who were intermittently exposed to opioids exposure to high doses (180 mg or more of oral morphine equivalent) occurred at some point in 2,095 (4%) subjects and 1,257 (2.6%) were exposed to very high doses (300 mg or more of oral morphine equivalent).

In subjects who were continuously exposed to opioids, 7.6% were exposed to high doses of opioids and 2.9% were exposed to very high doses of opioids at some point. Ten percent of subjects who were continuously exposed to opioids with a cancer diagnosis were exposed to high doses of opioids compared with 7% of subjects who were continuously exposed to opioids without a cancer diagnosis.

In subjects intermittently exposed to opioids, 18.7% reached doses of 100 mg or more of oral morphine equivalent. In subjects continuously exposed to opioids, 19.9% reached doses of 100 mg or more of oral morphine equivalent.

## Discussion

The present study reports the patterns of opioid use in a large diverse population across the United States over a substantial time period. Such data are difficult to obtain in traditional clinical studies, but are readily available in pharmacoepidemiologic database studies.

The study showed that intermittent exposure to opioids is a common phenomenon, a finding that has been described previously [18], and that in subjects with intermittent exposure, the dose of opioids remains stable over time. This group potentially includes subjects with subacute pain, pain exacerbations and chronic pain.

The initial median daily oral morphine equivalent dose was approximately 50 mg. Such doses aligned with those reported in studies performed in chronic pain clinics [19] and in the general population [18]. In the latter study, Von Korff et al described opioid use in noncancer patients in 2 US health plans.

In subjects with no cancer diagnosis and continuous exposure to opioids, the 95^th ^percentile dose rose early, but the mean, 25^th^, 50^th^, and 75^th ^percentile doses remained stable for the first 2 years of use then increased. However, over the full eight year course of the study the median dose of 45 mg increased to 130 mg and the 95^th ^percentile dose increased from 142 mg to 210 mg. Seven percent of subjects with no cancer diagnosis received at some point in time high doses of opioids.

The results of the present study are similar to the study by Bercovitch et al in patients receiving palliative care for terminal illnesses. These authors found that only a small percentage of subjects (approximately 9%) required doses of morphine equivalent to 300 mg or more [16]. The study by Sullivan et al, although not directly assessing the variation of opioid dose over time, corroborates our findings [2]. In that study, opioid use was characterized in commercially insured and publicly insured populations over a 6-year period. The study found that the cumulative yearly opioid dose increase was due to increases in the number of days supplied rather than the dose per day supplied [2].

The very large number of subjects exposed to opioids intermittently permits us to characterize with confidence the dose of opioids over time. On the other hand, only a small number of subjects were continuously exposed to opioids for more than 4 years. Therefore, extrapolating the findings of the study beyond this time period is not recommended.

Dose escalation is considered one of the major factors that could curtail the effectiveness of opioids [1,10]. The findings of this study show that dose escalation among commercially insured patients who are prescribed opioids continuously occurred in seven percent of subjects. For most subjects with continuous exposure, dose escalation was seen only after the first 2 years of use. A study in a different population, subjects with back injuries at risk for long term disability continuously exposed to opioids for a year, found a more rapid dose escalation --thirty nine percent of subjects moved to a higher dose category at the last quarter of follow up [20].

Exposure to opioids could lead to serious adverse events. A recent study that assessed the risk of overdose in subjects who had continuously received opioids for chronic non-cancer pain for at least three months found overdoses among 0.5% of subjects (1.5 events/1,000 person years of follow-up), and observed that subjects receiving 100 mg or more of morphine had a nine-fold increase in overdose risk [17]. In the present study approximately twenty percent of the subjects received such doses, at some time during treatment. Patients receiving these doses need close supervision. Future research should attempt to better define the patient groups in which prescription of opioids for non-cancer pain is safe, and those in which it is not.

Among the limitations of the present study, are: the PharMetrics Patient-Centric database reflects the commercially insured population and, therefore, overrepresents the healthy population which is able to work and underrepresents individuals 65 years or older and disabled and low income populations which are insured through Medicare or Medicaid programs. These limitations could affect the generalizability of the study findings. Attrition of subjects because of loss of health insurance may also affect a study like the present one, that is based on claims data, but some reassurance about this possible source of bias is provided by the finding doses in the preceding six months were similar for subjects who left the cohort and those who remained in it. In addition, the claims database reflects only those dispensings that were submitted for reimbursement in the outpatient setting. It is not able to identify medications that a patient may have obtained outside the health care benefit system. However, the PharMetrics Patient-Centric database captures data from all retail and mail-order dispensing regardless of which health care provider in the plan issues the prescription.

Pain intensity or pain relief information is absent in claims databases. Therefore, it is unknown whether subjects stopped treatment with opioids because of disease improvement, the lack of efficacy, or the lack of tolerability. Systematic reviews have found that up to one third of patients in clinical trials withdraw because of opioid adverse events and up to 18% of patients withdraw because of inadequate pain relief [3,5].

The inability to link with certainty the opioid dispensings directly to the diagnoses that they were intended to address, and the fact that PharMetrics uses the ICD-9 system to code the medical diagnosis, precluded the use of a pain taxonomy based on the pain mechanism. Thus, no attempts were made to compare opioid dose across pain categories, with the exception of the presence of cancer diagnosis.

The pattern of dose escalation observed in this study could be a reflection of the success of the adequate patient selection and careful evaluation by health care providers when prescribing opioids. Consequently, physicians and other health care providers should continue following the principles of good medical practice, and develop treatment plans tailored to the individual and the presenting problem when prescribing opioids, as recommended by many pain organizations such as the American Academy of Pain Medicine and the American Pain Society [21].

## Conclusions

Dose escalation is uncommon in subjects with intermittent exposure to opioids.

For subjects with continuous exposure to opioids who have cancer, doses rise substantially with time. For those without cancer, doses remain relatively stable for the first 2 years of use, but subsequently increase. Seven percent of subjects with no cancer diagnosis will be exposed to daily doses of 180 mg or more of morphine equivalent at some point

## Competing interests

M. Soledad Cepeda, Mila Etropolski, Rachel Weinstein, Daniel Fife, and Amy Matcho are employees of Johnson & Johnson Pharmaceutical Research & Development. Johnson & Johnson Pharmaceutical Research & Development is an affiliate of Ortho-McNeil-Janssen Pharmaceuticals, Inc, which markets several analgesic drug products including opioids and over-the-counter analgesics such as acetaminophen.

## Authors' contributions

MSC: conceived of the study, participated in its design, execution, and interpretation of data, and drafted the manuscript. ME: participated in the design of the study, interpreted the data, and critically revised the manuscript. RW: participated in the design and execution of the study and critically revised the manuscript. DF: participated in the design of the study, interpreted the data, and critically revised the manuscript. RB: performed the statistical analysis, interpreted the data, and critically revised the manuscript. AM: performed the programming to create the analytic data set, participated in the interpretation of the data and critically revised the manuscript. All authors read and approved the final manuscript.

## Pre-publication history

The pre-publication history for this paper can be accessed here:

http://www.biomedcentral.com/1472-684X/9/14/prepub

## References

[B1] Ballantyne JC Mao J Opioid therapy for chronic pain N Engl J Med 2003 349 1943 1953 10.1056/NEJMra025411 14614170

[B2] Sullivan MD Edlund MJ Fan MY Devries A Brennan BJ Martin BC Trends in use of opioids for non-cancer pain conditions 2000-2005 in Commercial and Medicaid insurance plans: the TROUP study Pain 2008 138 440 449 10.1016/j.pain.2008.04.027 18547726 PMC2668925

[B3] Furlan AD Sandoval JA Mailis-Gagnon A Tunks E Opioids for chronic noncancer pain: a meta-analysis of effectiveness and side effects CMAJ 2006 174 1589 1594 16717269 10.1503/cmaj.051528 PMC1459894

[B4] Deshpande A Furlan A Mailis-Gagnon A Atlas S Turk D Opioids for chronic low-back pain Cochrane Database Syst Rev 2007 CD004959 17636781 10.1002/14651858.CD004959.pub3

[B5] Noble M Tregear SJ Treadwell JR Schoelles K Long-term opioid therapy for chronic noncancer pain: a systematic review and meta-analysis of efficacy and safety J Pain Symptom Manage 2008 35 214 228 18178367 10.1016/j.jpainsymman.2007.03.015

[B6] Cepeda MS Camargo F Zea C Valencia L Tramadol for osteoarthritis: a systematic review and metaanalysis J Rheumatol 2007 34 543 555 17343302

[B7] Eisenberg E McNicol E Carr DB Opioids for neuropathic pain Cochrane Database Syst Rev 2006 3 CD006146 16856116 10.1002/14651858.CD006146

[B8] Martell BA O'Connor PG Kerns RD Becker WC Morales KH Kosten TR Fiellin DA Systematic review: opioid treatment for chronic back pain: prevalence, efficacy, and association with addiction Ann Intern Med 2007 146 116 127 17227935 10.7326/0003-4819-146-2-200701160-00006

[B9] Chu LF Angst MS Clark D Opioid-induced hyperalgesia in humans: molecular mechanisms and clinical considerations Clin J Pain 2008 24 479 496 10.1097/AJP.0b013e31816b2f43 18574358

[B10] Cepeda MS Should opioids be prescribed to treat patients with osteoarthritis? Nat Clin Pract Rheumatol 2008 4 180 181 10.1038/ncprheum0758 18268548

[B11] Suissa S Garbe E Primer: administrative health databases in observational studies of drug effects--advantages and disadvantages Nat Clin Pract Rheumatol 2007 3 725 732 10.1038/ncprheum0652 18037932

[B12] Jordan KP Croft P Opportunities and limitations of general practice databases in pain research Pain 2008 137 469 470 10.1016/j.pain.2008.04.015 18485597

[B13] Berger A Hoffman DL Goodman S Delea TE Seifeldin R Oster G Therapy switching in patients receiving long-acting opioids Ann Pharmacother 2004 38 389 395 10.1345/aph.1D109 14742831

[B14] Hall GC Carroll D Parry D McQuay HJ Epidemiology and treatment of neuropathic pain: the UK primary care perspective Pain 2006 122 156 162 10.1016/j.pain.2006.01.030 16545908

[B15] Arellano FM Yood MU Wentworth CE Oliveria SA Rivero E Verma A Rothman KJ Use of cyclo-oxygenase 2 inhibitors (COX-2) and prescription non-steroidal anti-inflammatory drugs (NSAIDS) in UK and USA populations. Implications for COX-2 cardiovascular profile Pharmacoepidemiol Drug Saf 2006 15 861 872 10.1002/pds.1343 17086563

[B16] Bercovitch M Adunsky A Patterns of high-dose morphine use in a home-care hospice service: should we be afraid of it? Cancer 2004 101 1473 1477 10.1002/cncr.20485 15368335

[B17] Dunn KM Saunders KW Rutter CM Banta-Green CJ Merrill JO Sullivan MD Weisner CM Silverberg MJ Campbell CI Psaty BM Von KM Opioid prescriptions for chronic pain and overdose: a cohort study Ann Intern Med 2010 152 85 92 20083827 10.1059/0003-4819-152-2-201001190-00006 PMC3000551

[B18] Korff MV Saunders K Thomas RG Boudreau D Campbell C Merrill J Sullivan MD Rutter CM Silverberg MJ Banta-Green C Weisner C De facto long-term opioid therapy for noncancer pain Clin J Pain 2008 24 521 527 10.1097/AJP.0b013e318169d03b 18574361 PMC3286630

[B19] Buntin-Mushock C Phillip L Moriyama K Palmer PP Age-dependent opioid escalation in chronic pain patients Anesth Analg 2005 100 1740 1745 10.1213/01.ANE.0000152191.29311.9B 15920207

[B20] Franklin GM Rahman EA Turner JA Daniell WE Fulton-Kehoe D Opioid use for chronic low back pain: A prospective, population-based study among injured workers in Washington state, 2002-2005 Clin J Pain 2009 25 743 751 10.1097/AJP.0b013e3181b01710 19851153

[B21] A consensus statement from American Academy of Pain Medicine and American Pain Society. The use of opioids for the treatment of chronic pain: a consensus statement from American Academy of Pain Medicine and American Pain Society 2008 http://www.ampainsoc.org/advocacy/opioids.htm 12-29-2008.

